# Interictal aggression in rats with chronic seizures after an early life episode of status epilepticus

**DOI:** 10.1002/epi4.12734

**Published:** 2023-03-31

**Authors:** Lucie Suchomelova, Kerry W. Thompson, Roger A. Baldwin, Jerome Niquet, Claude G. Wasterlain

**Affiliations:** ^1^ Department of Neurology David Geffen School of Medicine at UCLA Los Angeles California USA; ^2^ Neurology Research (151) VA Greater Los Angeles Healthcare System Los Angeles California USA; ^3^ Department of Biology Occidental College Los Angeles California USA

**Keywords:** aggression, seizures, status epilepticus

## Abstract

**Objective:**

In spite of anecdotal reports describing an association between chronic epilepsy and interictal aggressiveness, and of a few studies suggesting that such an association is common in temporal lobe epilepsy, this concept has not been generally accepted by epileptologists. In the course of studies of the long‐term consequences of limbic status epilepticus (SE) in juvenile rats, we noticed that experimental animals, unlike littermate controls, could not be housed together because of severe fighting. We now report a study of interictal aggression in those rats.

**Methods:**

Long‐term behavioral consequences of lithium/pilocarpine SE were studied 3 months after SE had been induced with lithium and pilocarpine in male Wistar rats at age 28 days. Chronic spontaneous seizures developed in 100% of animals. We tested rats for territorial aggression under the resident‐intruder paradigm. We measured the number of episodes of dominance (mounting and pinning), and agonistic behavior (attacks, boxing, and biting).

**Results:**

Untreated lithium/pilocarpine SE induced a large increase in aggressive behavior, which involved all aspects of aggression in the resident‐intruder paradigm when tested 3 months after SE. The experimental rats were dominant toward the controls, as residents or as intruders, and showed episodes of biting and boxing rarely displayed by controls. They also displayed increased aggressiveness compared with controls when tested against each other.

**Significance:**

This robust model offers an opportunity to better understand the complex relationship between seizures, epilepsy, and aggression, and the role of age, SE vs. recurrent spontaneous seizures, and focal neuronal injury in the long‐term behavioral effects of SE.

**Key points**

- Untreated LiCl/pilocarpine status epilepticus (SE) in rats induced a large increase in aggressive behavior when tested 3 months after SE.
- The experimental rats were dominant toward controls and showed increased agonistic behavior (attacks, boxing, and biting).
- When post‐SE experimental rats were tested together, they expressed prominent agonistic behavior but did not display dominant behavior.
- This model is a useful tool in the study of aggressive behavior associated with spontaneous recurrent seizures.

## 1 INTRODUCTION

In humans, the relationship between epilepsy and aggression has a long and controversial history, and includes postictal aggression^1^, ^2^, ^3^; aggression during postictal psychosis;^4^, ^5^, ^6^, ^7^ the use of epilepsy as a defense in criminal trials;^8^ ictal aggression in frontal lobe or other focal epilepsy;^9^ aggression as part of the rare behavioral syndrome associated with temporal lobe epilepsy^10^, ^11^, ^12^, ^13^, ^14^ or of the controversial “epileptic personality;”^15^, ^16^, ^17^ and anecdotal reports of aggression in patients with chronic epilepsy.^18^ However, linkages of epilepsy to each one of these types of aggression, if they exist, are complex and may inadvertently stigmatize people with epilepsy if they are not fully understood or described.

The study of human aggression using animal seizure models has many potential pitfalls,^19^ but there a number of studies that show common neuroanatomical,^20^, ^21^, ^22^, ^23^, ^24^ neurochemical,^9^, ^25^ and genetic substrates^26^ for epilepsy and aggression, which can be investigated using basic models. In particular, the kindling model of epilepsy^27^, ^28^ and spontaneous temporal lobe seizures following the induction of status epilepticus (SE)^20^, ^29^ have been used to study different forms of aggression in rats using validated and quantifiable methods. Some of these reports support a connection between seizure history and con‐specific aggression in different social contexts.^30^ These types of studies may shed light on the neural substrates of aggression in the background of epilepsy.

In our own studies, during long‐term seizure monitoring or behavioral studies following SE induced by lithium and pilocarpine in juvenile or adult rats, we had to house experimental rats individually rather than the customary four animals per cage, because of a high incidence of unusual aggressive behavior. Agonistic behaviors between cage mates were observed frequently in group cages. In “extreme” cases,^31^ some rats were found with signs of trauma around their head and neck areas, which is quite rare in control rats housed four per cage. To characterize these behavioral changes, we conducted a pilot study of aggression in rats subjected to SE during the juvenile period (Post Natal Day [PND] 28) and tested for behavioral changes 3 months later. This study revealed significantly elevated aggressive behavior in post‐SE animals compared with controls not subjected to SE.

## 2 METHODS

### 2.1 Animals

Male Wistar albino rats (Simonsen Lab) were used on PND28. The day of birth was considered as Day 0. Pups were weaned at PND21. All animals were housed in a temperature‐ and humidity‐controlled room with 12‐h light–dark cycles (the dark cycle starts at 6 pm) and had free access to food and water. All experiments were conducted with the approval and in accordance with the regulations of the Institutional Animal Care and Use Committee of West Los Angeles VA Medical Center. The protocol number was 97110‐4. We used six animals per group for the acute EEG study and eight animals per group for the territorial aggression test and chronic EEG monitoring.

### 2.2 Induction of SE and acute EEG monitoring

**FIGURE 1:**
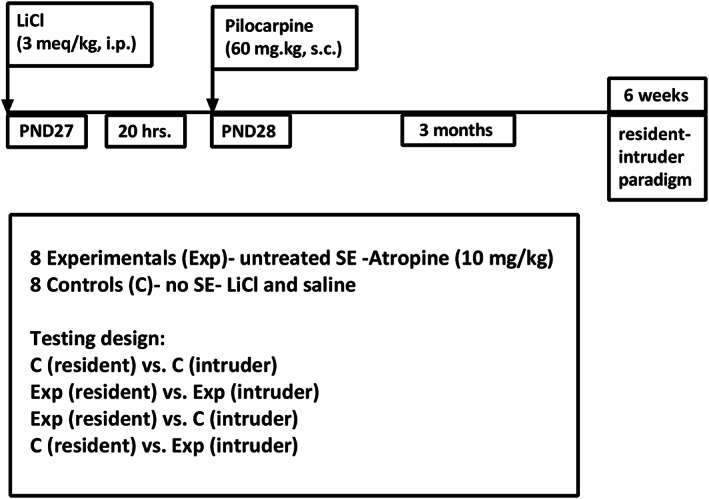
Design and timeline of territorial aggression experiment in lithium–pilocarpine SE‐induced seizures in PND28 rats, 3 months after SE. Each animal was tested as a resident and intruder. Over a period of 6 weeks, groups were tested with controls vs. controls, experimentals vs. experimentals, and controls vs. experimentals. No two animals were paired more than once, and individual animals were tested every fourth day. Abbreviations: PND28, postnatal day 28; SE, status epilepticus.

SE was induced with lithium and pilocarpine.^32^ Lithium (3 mE/kg) was administered intraperitoneally on the day of surgery. The detailed surgical method is described in Suchomelova et al. (2006).^32^ Briefly, the animals were anesthetized with halothane using a vaporizer. A tripolar electrode was then connected to skull screws (the first two were inserted into the skull above right and left frontal cortex, the third one was placed over the cerebellum). Twenty hours later, a subcutaneous injection of pilocarpine (60 mg/kg) was administered (Figure 1). We used a video‐EEG system for 24 h continuous monitoring. The EEG onset of first ictal activity and continuous polyspike activity were measured. The experimental group was treated at 70 min after pilocarpine with atropine in amounts sufficient to block most effects of pilocarpine (10 mg/kg), in order to remove the original trigger that might re‐start seizures treatment stopped them. Atropine did not alter the seizure course in any animal, demonstrating that seizures were no longer dependent on the original muscarinic trigger.^32^ No other anti‐seizure medication was given to stop the seizures. The “no SE” control group received only lithium and equal amounts of vehicle. All animals were rehydrated with saline approximately 5 h after SE (10% of body weight, s.c.).

The course of SE in atropine‐treated animals was described in detail in Suchomelova et al. (2006).^32^ Briefly, pilocarpine induced the first spikes in 5.2 ± 2.3 min, the first discrete seizure after 8.2 ± 2.1 min and continuous polyspike activity after 15.5 ± 2.5 min. Continuous polyspike activity was replaced, after 185.7 ± 21.1 min, by multiple intermittent seizures 65.4 ± 19.3 during 24 h of continuous recording. The duration of SE was 1087.7 ± 86.2 min after onset of SE. The “no SE” animals did not develop SE and had a mean spike frequency of 3.6 ± 0.7 spikes/h.

### 2.3 Behavioral testing

For resident‐intruder testing, a separate group of animals undergoing SE at PND28, but not implanted with electrodes (experimentals), and vehicle‐injected controls were tested 3 months later for territorial aggression. Animals were individually housed in transparent plastic cages for 10 days prior to behavioral testing to ensure home cage familiarity. We tested rats for territorial aggression under the modified resident‐intruder paradigm^29^, ^33^, ^34^: An “intruder” rat is introduced into the cage of a “resident” rat for a 15 min session. Aggression tests were administered strictly once every 4th day during the dark period at 7 pm (6 pm marked the beginning of the dark cycle). At the beginning of each session, the resident rat in its home cage was positioned in the recording site and given 5 min to accustom to testing conditions. Subsequently, an unfamiliar intruder rat was introduced into the same cage for a 15 min video‐recorded session. A clean transparent cage was inverted on top of the testing cage (as a lid) to allow additional space for movement but to prevent escape. Each animal was tested only once per day to prevent the decrease in responsiveness due to fatigue and/or habituation to the resident‐intruder scenario. Additionally, no animal was tested more than twice per week to reinforce home cage familiarity and fresh/instinctive reactions to intruders. Control rats were tested against other controls both as residents and intruders to establish their baseline behavior. Experimental rats were tested against controls and against other experimentals as both resident and intruders (Figure 1). No two animals were tested together more than once to exclude behavior due to any pre‐established dominance. We measured the number of episodes of dominant mount (ventral/dorsal contact and immobilization of opponent), pinning (ventral/ventral contact after opponent is forced onto its back and immobilized in position), and agonistic behavior: attacks (lunging onto opponent), boxing (upright stance, facing opponent, pushing opponent with forepaws), and biting (direct dental contact of one rat with another). Animals demonstrating a convulsive seizure during the 5 min acclimation period prior to testing, or during testing, were excluded from the testing for that session. Five out of the eight animals expressed at least one convulsive seizure prior to, or during, testing.

### 2.4 Chronic EEG/video monitoring

Immediately after territorial aggression testing, the animals were anesthetized and implanted with magnetically activated implants for transmitting EEG signals.^32^ Briefly, the implant cables were connected to skull screws placed over the left frontal and occipital cortex under Xylazine (15 mg/kg)/Ketamine (60 mg/kg) anesthesia. After 1 week of postoperative recovery, the animals were monitored for 1 week. EEG/video results are not discussed in detail in this paper.

### 2.5 Statistical analysis

In the territorial aggression study, each group (controls and experimentals) consisted of eight animals. The number of episodes in the text is represented as a Minimal/Maximal/Median value. The dataset in the figures is represented by box and whisker plots. The central lines within the box represent medians, the two ends of the rectangle represent first and third quartiles. The upper and lower whiskers extend to the lowest and highest value in the dataset. Each filled circle represents the number of episodes each animal expressed during 15 min of the resident‐intruder testing.

Statistical comparisons were made using Mann–Whitney *U*‐test for nonparametric data using GraphPad statistical software (Dotmatics).

## 3 RESULTS

### 3.1 Spontaneous seizures

Post‐SE animals that were used in the territorial aggression study were evaluated for spontaneous seizures following the behavioral observations. All of the experimental animals displayed convulsive spontaneous recurrent seizures, with an average seizure frequency of 17.5 ± 5.1/day and a mean seizure duration of 26.9 ± 7.3 s.

### 3.2 Control vs. control

**FIGURE 2:**
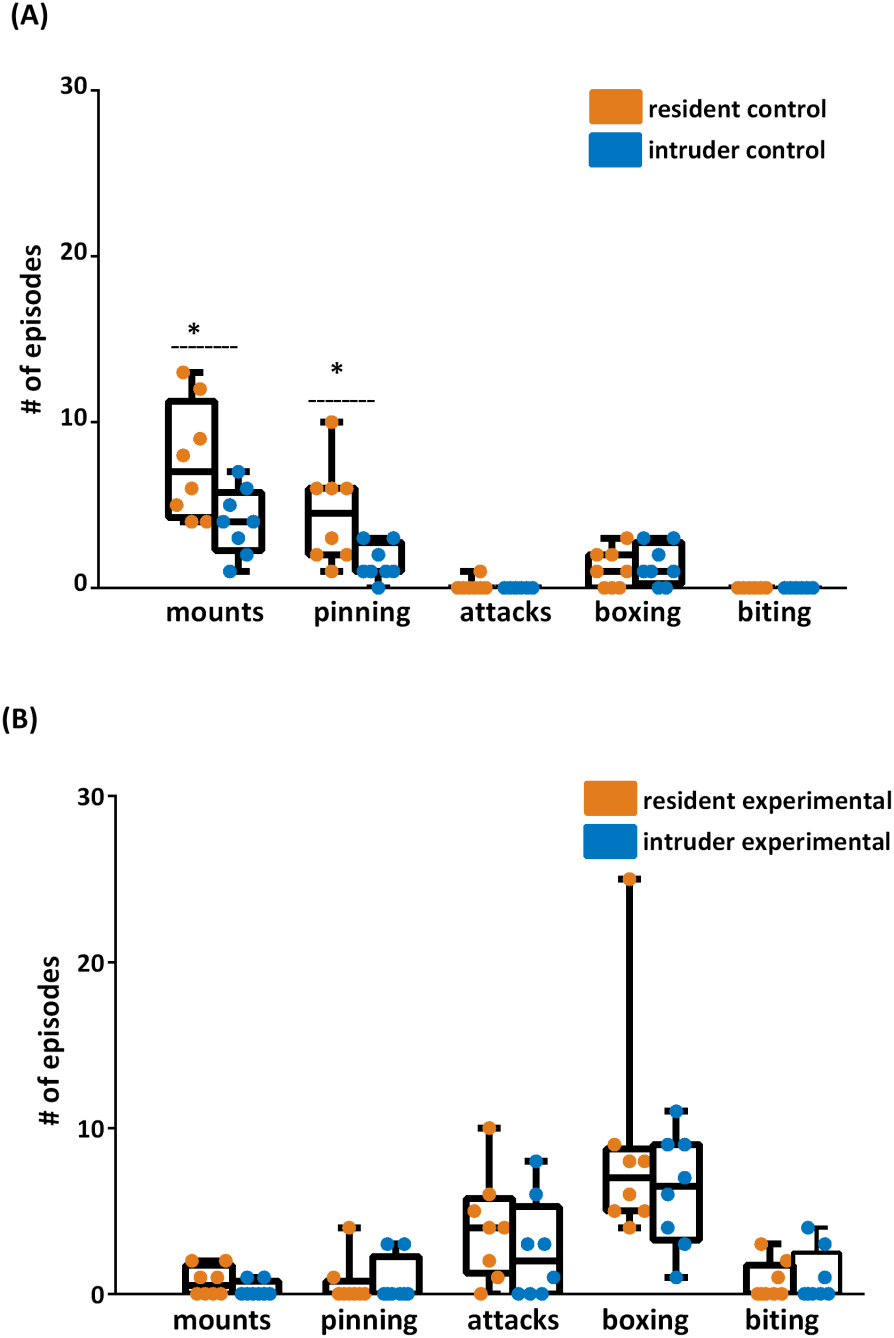
Aggressive behavior differed between control and experimental animals when tested under the resident‐intruder paradigm 3 months after SE. (A) Resident controls expressed more dominant behavior (mounts and pinning) when exposed to intruder controls. Agonistic behavior (attacks, boxing, and biting) was nonexistent or did not differ between residents and intruders. (B) Experimental animals tested together displayed less dominant and more agonistic behavior. Neither dominant nor agonistic behavior differed between residents and intruders. Statistics: Mann–Whitney U test, *p < 0.05, resident controls vs intruder controls, resident experimentals vs. intruder experimentals, *n* = 8 rats per group.

To establish the baseline behavior in the resident‐intruder paradigm, we first exposed control animals to each other. The control residents displayed dominant behavior with a significant increase in dominant mounts (4.0/13.0/7.0 vs. 1.0/7.0/4.0) and pinning behavior (1.0/10.0/4.5 vs. 0.0/3.0/1.0) compared with intruders. Agonistic behavior was minimal, with no attacks, no biting, and only a small amount of boxing behavior (Figure 2A).

### 3.3 Experimental vs. experimental

The experimental rats, when tested together, showed prominent agonistic behavior in both the resident and the intruder groups. Attacks were frequent, boxing was common and often long‐lasting, and biting was relatively common. Surprisingly, they often did not display more traditional dominant behavior (dominant mounts and pinning) as residents when compared to the intruders. There were no statistical differences between residents and intruders in any of the observed behaviors (Figure 2B).

### 3.4 Experimental vs. control

**FIGURE 3:**
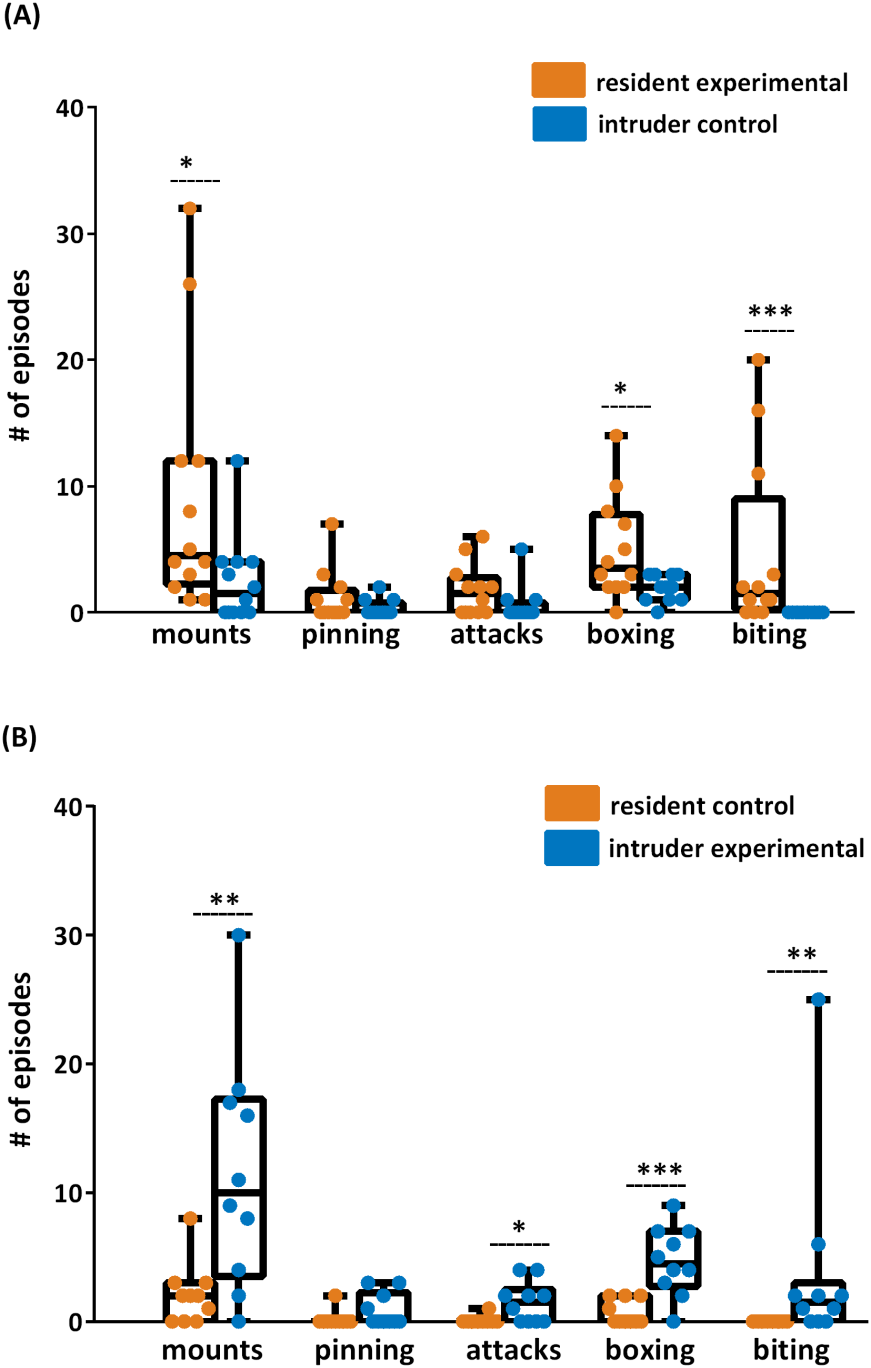
Experimental animals were more dominant and showed more agonistic behavior when tested with controls. (A) Experimental residents expressed a large amount of dominant (mounts) and agonistic (boxing and biting) behavior when tested with control intruders. (B) Similarly, experimental intruders when tested with control residents displayed more dominant (mounts) and agonistic behavior (attacks, boxing, and biting) than resident controls. Statistics: Mann–Whitney U‐test, *p < 0.05, **p < 0.01, ***p < 0.001, residents vs. intruders, *n* = 8 rats per group.

When exposed to intruder controls, the resident experimental animals expressed the expected dominant behavior with a large excess of dominant mounts (1.0/32.0/4.5 vs. 0.0/12.0/1.5) and a non‐significant trend toward increased numbers of pinning. They also displayed all aspects of agonistic behavior. We observed an increased number of boxing episodes (0.0/14.0/3.5 vs. 0.0/3.0/2.0) and episodes of biting the opponent (0.0/20.0/1.5 vs. 0.0/0.0/0.0; Figure 3A).

However, when intruder post‐SE rats were exposed to resident controls, there was a dramatic reversal of the expected resident versus intruder hierarchy. Post‐SE intruders showed a large excess of dominant mounts over resident controls (0.0/30.0/10.0 vs. 0.0/8.0/2.0). They also showed prominent agonistic behavior when compared to control residents (0.0/4.0/1.5 vs. 0.0/1.0/0.0 attacks, 0.0/9.0/4.5 vs. 0.0/2.0/0.0 boxing, and 0.0/25.0/1.5 vs. 0.0/0.0/0.0 biting; Figure 3B).

**FIGURE 4:**
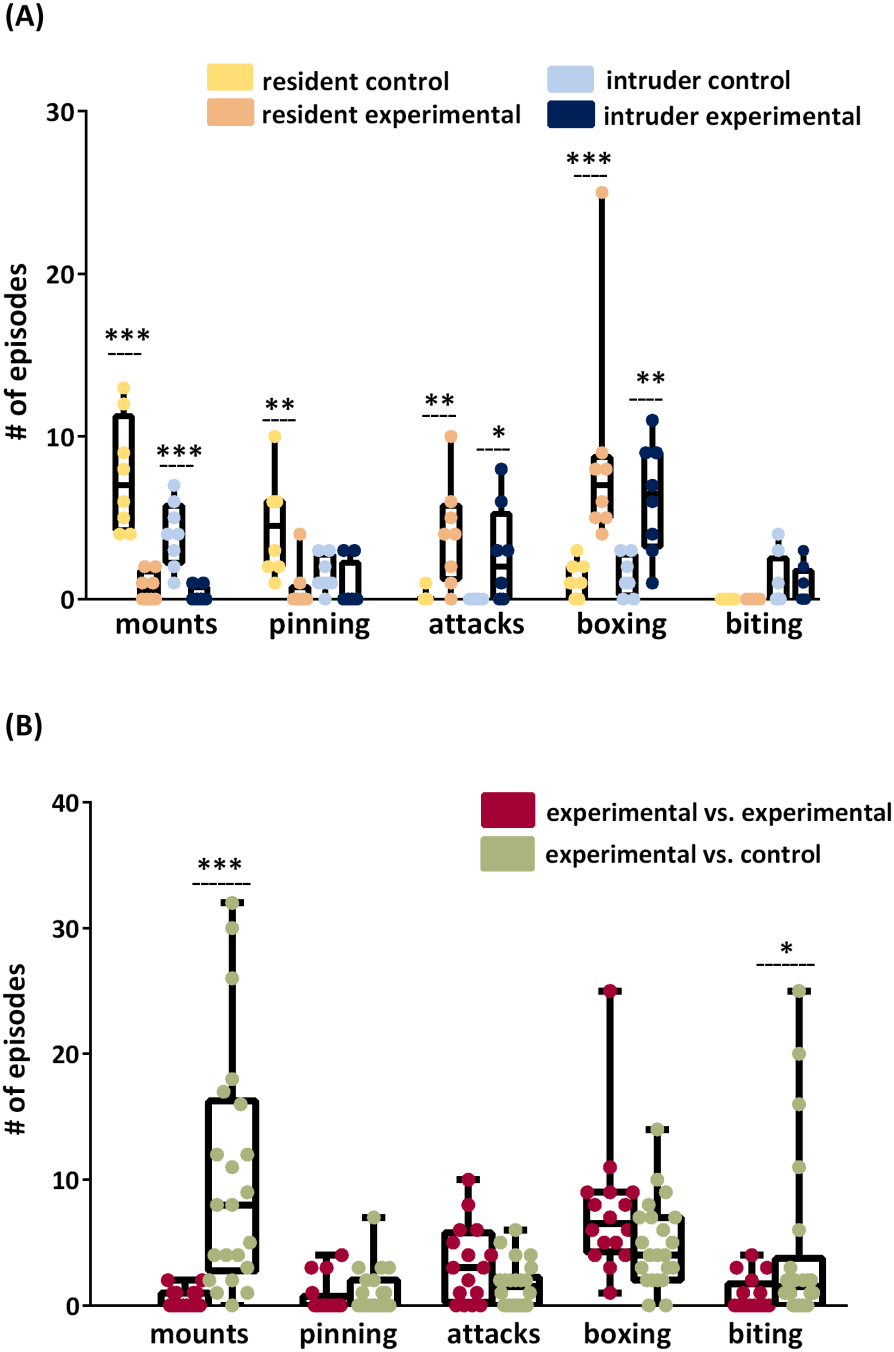
(A) Overall, resident post‐SE animals (as residents and also as intruders) showed significantly less dominant (mounts and pinning) and more agonistic behavior (attacks and boxing) when compared to the behaviors of resident or intruder control animals. (B) Pooled data from all post‐SE animals showed increased dominance (mounts) and agonistic behavior (biting) against controls when compared to other experimentals. Statistics: Mann–Whitney U‐test, *p < 0.05. Statistics: Mann–Whitney U‐test, *p < 0.05, **p < 0.01, ***p < 0.001. (A) resident controls vs. resident experimentals, intruder controls vs. intruder experimentals. (B) experimentals with experimentals vs. experimentals with controls. *n* = 8 rats per group.

Overall, resident post‐SE animals showed significantly less dominant (0.0/2.0/0.5 vs. 4.0/13.0/7.0 episodes of mounts and 0.0/4.0/0.0 vs. 1.0/10.0/4.5 pinning episodes) and more agonistic behavior (0.0/10.0/4.0 vs. 0.0/1.0/0.0 attacks and 4.0/25.0/7.0 vs. 0.0/3.0/1.0 boxing episodes) when compared to the behavior of control animals (Figure 4A). As intruders, post‐SE rats also showed less dominant (mounts) and more agonistic behavior (attacks and boxing) when compared to the behavior of control animals (Figure 4A).

When we pooled resident and intruder data, the experimental rats showed more dominant behavior toward controls than toward other experimentals (0.0/32.0/8.0 vs. 0.0/2.0/0.0 mounts) and showed increased biting (0.0/25.0/1.5 vs. 0.0/4.0/0.0) regardless of their resident or intruder status (Figure 4B).

## 4 DISCUSSION

The current study was prompted by the incidental observation of an increase in the frequency and nature of fighting in post‐SE animals. It confirmed that post‐SE animals showed increased aggression in the form of increased dominant mounts, even when they were the intruder in the cage. Furthermore, agonistic aggression such as sudden attacks with boxing and biting were significantly increased after SE. Changes in aggressive behavior were long‐lasting and persisted into the chronic epilepsy period several months after SE. Differences between post‐SE animals and controls were dramatic and may provide a reliable model for the study of the poorly understood relationship between seizures, epilepsy, and aggression.

Increased aggression has been reported in previous studies following lithium–pilocarpine seizures. Persinger et al. (1993)^22^ observed head and neck lesions after SE, and Desjardins et al. (2001)^30^ reported that when rats were housed in groups of six, the amount of biting and boxing increased with the proportion of post‐SE animals in the group. However, Smolensky et al. (2019),^35^ using a similar model with SE at 7 weeks, found only minor changes in the resident‐intruder paradigm.

We do not know whether this increased aggression, deep into the chronic seizure period, is age‐ or model‐specific. Although we have witnessed signs of aggression after SE was induced in adults, the severity of changes in the current study after SE at postnatal day 28 raises the question of the age dependence of this finding. The role of the initial episode of SE versus that of spontaneous recurrent seizures in behavioral changes is also unknown. Previous studies have described the development of post‐SE agonistic aggression coming to an asymptote in the month following lithium–pilocarpine seizures initiated in adult rats.^22^ Our unpublished observations in adult rats subjected to SE are consistent with those earlier results, but here, we show that seizure‐induced brain alterations occurring earlier in life (PND28) produce substantially elevated, and chronic, aggression in rats.

Previous investigators^22^, ^30^ used rats between 90 and 120 days old, gave lithium orally, used a slightly lower amount of pilocarpine (30 vs. 60 mg/kg), and injected acepromazine after seizure onset, and the effects of these changes on seizure severity are unknown. A study of kainic acid‐induced SE in rats did not find increased aggression after SE.^29^ However, a study of cats after intratemporal kainic acid SE found increased emotionality, with aggression, in post‐SE cats.^36^ Studies of rodents with kindled seizures showed only mild changes^28^, ^37^, ^38^ This heterogeneity of outcomes suggests that some measure of model specificity exists, but gives little clue regarding the key differences between models that generate postictal aggression and those that do not. We used a model of relatively severe SE and did not treat seizures at 2 h with diazepam, and this could be a factor. It is well established that a longer duration of SE alters the long‐term histopathological consequences.^39^, ^40^, ^41^, ^42^ SE‐induced neuronal injury, and probable synaptic reorganization, may play an important role in the development and expression of aggressive behavior.^30^, ^43^ Neurophysiological substrates mediating aggression have been studied using both electrical stimulation and ablation^44^, ^45^, ^46^ of targeted pathways. Limbic areas such as the amygdala have been linked to aggression in these studies. It is well known that the amygdala and synaptically connected areas, such as the hippocampus, are also involved in limbic seizure induction as well as the resulting behavioral outcomes.^37^, ^47^, ^48^ Importantly, several seizure models have been used to study aggression^27^, ^29^, ^31^, ^38^, ^47^ with varied results.

The human relevance of these changes remains to be explored. The current seizure model is not relevant to the problems of postictal or ictal aggression,^1^, ^2^, ^3^, ^9^ of postictal psychosis,^4^, ^5^, ^6^, ^7^ or of the use of epilepsy as a legal defense.^8^ It is probably not relevant to the “Epileptic personality,”^15^, ^16^, ^17^ since most epileptologists do not believe that such a personality is a feature of epilepsy. However, it might become a useful tool to study the relationship between epilepsy and aggression in the rare behavioral syndrome associated with chronic poorly controlled epilepsy^10^, ^11^, ^12^, ^13^, ^14^ and in anecdotal reports of aggression in patients with epilepsy.^18^, ^19^

In summary, lithium–pilocarpine SE in juvenile rats leads to spontaneous seizures and increased agonistic aggression. The model lends itself to the study of SE‐associated changes in aggressive behavior, of the relationship between behavioral and anatomical changes following SE in the chronic seizure period, and of the influence of treatment on the development, and possibly the prevention, of behavioral sequalae of SE. Studies like these could potentially shed light on the neurobiological substrates of ictal vs interictal aggression, and discern the impact of important complicating factors such as the effects of medication and brain damage on multiple forms of aggression.

## AUTHOR CONTRIBUTIONS

All co‐authors have made substantial intellectual/conceptual contributions to the work. Lucie Suchomelova participated in experimental design, data acquisition, analysis, and interpretation of data. She also participated in drafting and critically revising the article. Kerry Thompson participated in analysis and interpretation of the data and in drafting and critically revising the article. Roger Baldwin participated in experimental design and data acquisition. Jerome Niquet participated in data acquisition, analysis, and interpretation of data. Claude Wasterlain participated in experimental design, in drafting and critically revising the article, and in the final approval of the manuscript version to be published.

## CONFLICT OF INTEREST STATEMENT

The authors report no conflict of interest. The authors confirm that they have read the Journal's position on issues involved in ethical publication and affirm that this report is consistent with those guidelines.
